# Supplementary material for: Sociocultural practices, beliefs, and myths surrounding newborn cord care in Bayelsa State, Nigeria: A qualitative study
Source: PLOS Glob Public Health. 2023 Mar 28;3(3):e0001299. doi: 10.1371/journal.pgph.0001299 (PMC10047526; doi:10.1371/journal.pgph.0001299)
Supplement: S1 Text — (DOCX) [file pgph.0001299.s001.docx]

**KNOWLEDGE, PRACTICE AND PREDICTORS OF GOOD NEWBORN CORD CARE AMONG MOTHERS IN BAYELSA STATE.**

**FOCUS GROUP DISCUSSION**

**Date of interview:** 12 – 05 – 2021

**Start time:** 9:28 am

**Stop time:** 9:59 am

**Interviewers name:** A.C.S.

**Note takers name:** O.R.E.

**Interview tool used:** Note pad and Sony IC Recorder

**Gender of Interviewee:** Eight Women

**LGA:** Emeya II Ogbia LGA, Bayelsa state.

Interviewees accepted to participate in the focus group discussion voluntarily and also agreed to have their voice recorded.

**Section 1: Introduction of Participants**

**Mother one**

Thank you very much. My name is **F.U.** I have been in Bayelsa for 6 years now. I have given birth to six children. I stop in Secondary school, I am ---- years old.

**Mother two**

My name is **A.B.A.** Am six years in Bayelsa. I stopped my education in secondary school I have 3 children, I am ---- years old.

**Mother three**

My name is **T.I.,** I have stayed in Emeyal over 11years now. I stopped in secondary school. I have four children, I am ---- years old.

**Mother four**

My name is **F.S.** I have been in Bayelsa for 9 months. I have three children. I am ---- years old.

**Mother five**

I am **J.I.** I’m from Bayelsa, so I’ve practically lived all my life in Bayelsa state. I have 4 kids and my education stopped at secondary school.

**Mother six**

My name is **W.O.** I’ve lived in this place for 13 years. I have 4 children. I am ---- years old.

**Mother seven**

My name is **J.P.** I have stayed in Bayelsa for 3 years and I have 1 child, I stopped at secondary school. I am ---- years old.

**Mother eight**

My name is **E.L.**, I’ve been in this community for more than fifteen years now and I have six children. I stop my school, secondary school.I am ---- years old.

**Where do mothers prefer to go to give birth in this community (Health centres or TBA)?**

**Response one**: Health centre

**Response two:** Most women prefer to deliver in eeeee in native place.

**Response three:** Most people prefer to deliver their baby at home …….native delivery.

**Why do they prefer this place of delivery?**

**Response one:** Some of them prefer to deliver at home because…….. Maybe they thought that if they go to the hospital it will be much expensive and some of them, prefer to go to the hospital because they have registered in the hospital so, they go there to deliver, some because they take the treatment with massaging people, that native way so they go back there to deliver .

**What are the common cultural practices adopted by mothers when taking care of their newborn?**

**Response one:**

Unnnnnn most people they use the native leaf to cut their baby’s cord, is the ‘African never die’……………….

**Interviewer: is there any special way they take care of the new born child?**

**Response all**: no no no there is no special way

**Are there any cultural myths or beliefs about the newborn’s cord?**

**Response one:**

Some child… when they give birth, some mothers did not cover the cord properly and allow air to enter, so it will worry the baby and the baby will start crying. Have been in emayah for fifteen years now, so I know the story, So some mothers will take the baby to a massaging …..or the one that knows the medicine to use and make a cross mark in the baby’s cord, so that the cord will not affect the baby

**Response two:**

That navel of a thing that she is talking about, the cross mark is when the baby have pain in the cord or strange noise by reason of infection, to stop the pain.

Right after a baby is born, how is the cord cut? Who does the cutting? What is usually used to cut the cord? Is anything done before the cord is cut? (Tying of cord)? What is usually used for the tying the cord?

**Response one:**

In the native side, they usually use razor blade en,,,, but at the hospital, as for me I don’t know oo because am always born in massaging place

**Response two:**

As for me it’s the nurse that deliver the baby but it’s at home … but she used blade.

**Who does the cutting?**

**Response one:**

the woman that delivered me the traditional woman that cut the cord.

**Response two:**

The nurse that delivered me cut the cord.

**What is usually used for the tying of the cord?**

**Response one**:

They use thread.

**Response two:**

In the hospital they use to use…… clip.

**Response three:**

We use raffia palm fronts to tie the cord.

**After birth, how is the cord usually cared for?**

**Response one:**

First of all, we use spirit lean the navel(cord) so that it will not have odor, the second one is that we use Salt, ‘Never die’, Ashes finish

**Response two:**

As for me I use, the nurse advise me to use eeee this thing spirit for the sake of odor, then she asked me to apply rob but my own understanding……………. I was with eeee one midwife so the woman taught me how to use never die,….. That, that was the best but later they asked me to use close –up that, that is the best that if you drop it two or three days, the cord will drop. That two or three days it will cut so i applied the spirit and is like the thing is delaying and the baby is crying, so I was feeling that the baby is feeling pain so I applied the close up the day I applied…… the close up from morning, afternoon and evening. Then the next day the cord fall off.

**Response four:**

Well after cutting the cord when you take the child home, What we usually do at home is to use hot water, we use hot water to press it but what I realy prefer is to face the baby down and use a cup so that the heat will go inside the belly of the child, then after that you use your,,,,,,, your spirit to press it and then,,,,,, after that you apply pepper then you also apply rob, then use alligator pepper to put it so that the hotness of the alligator pepper will enter inside the stomach, that process will not allow the child’s cord to pain and not to have issues.

**Interviewer: we heard some mothers use pepper?**

**Response one**: apart from alligator pepper we don’t know that one.

**Response:** all, is not pepper is alligator pepper

**Interviewer: the alligator pepper how is it used?**

**Response one:**

The alligator pepper you shew it with your mouth and put it in the belly (stomach) then you tie it and cover it

**What was your reason for using this method?**

**Response one:**

To avoid sickness to avoid contacting infection

**Response two:**

So that it will not make the cord to pain the child and also to avoid odor.

**The method you use does it make cord to fall off or separate quickly.**

**Response one:**

Is to fall off quickly and it will not delay because as for me when I first used spirit, the first woman that delivered my baby said that I should apply only spirit but when I see that it is delaying me I applied the other methods they thought me so that it will fall quickly.

**Was there any side effect?**

**Response one:**

NO

**Response two:**

If the cord fall off quick it use to affect the baby, that’s why the nurse they usually advise to use that ennnnn penicillin (clorhexidine) that one it will take like eight days before the cord will cut so I prefer the nurse advise the hospital own I don’t prefer the native

**What was the effect of the cord falling quickly?**

**Response three:** it’s like plantain, when you roast the platain in a fire as in fire that is not that hot, if you put platain the plantain will don in the up but not the inside, so if the navel fall quickly en the…….cord will heal outside and not inside so it will affect the baby. That’s why orefer the hospital method.

**Does anyone have experience of infected cord?**

**Response one:**

I don’t have experience but have seen a child that have that kind of cord problem as a result of applying different things, like most times the baby cord use to be black and it use to bleed most time, so it use to affect the baby, that one that the cord will fall off quick within three days sometimes the navel has not heal and close very well, there is still space that is why the baby cord use to bleed.

**What is usually used to prevent or treat problems with the baby’s cord?**

**Response one:**

I used rob, just rob and hot water

**Response two:**

Rob and hot water and now buy some syrup like Ampiclox, Vitamin C, and one other medicine but have forgotten the name. We use to apply this syrup so that it will heal the wound.

**Response three:**

Most at time the effects is that the water use to apply the baby cord is not hot enough it does not work effectively so I prefer boiling water so that it will heal the wounds.

**Who thought you how to use this methods?**

**Mother one**

From my mother

**Mother two**

From mothers

**Mother three**

From mother

**Mother four**

From the woman that delivered my baby, the massaging woman

**Mother five**

When you go for antenatal

**Mother six**

From mother

**Mother seven**

My mother and neighbor also

**Mother eight**

It’s normal, when you give birth to a child, mothers will be the first people to tell you. Then maybe in the hospital the midwife or the nurse

**Do you think this methods really work?**

**Response**: all affirmed with a Yes.

**Does the child cord heal better/improve**

**Response**: impro**ve**

**What kind of cord problem you heard or experienced.**

**Response one**:

Sometimes some of baby when air enter the cord the stomach will rise and will be making noise so they take the baby to the native place to treat

**Response two:**

Sometimes the midwife or nurses after birth use to cut the cord very short and it will make the baby to bleed and if you don’t take care of it very well it will kill baby.

**How common is this problem?**

**Response**: All mothers affirm is not common.

**Have you heard about some antiseptics (such as methylated spirit or chlorhexidine) which are commonly used to clean infant’s cords?**

**Response:** All mothers affirm yes.

**Which one is commonly used to clean the infants cord in this community ?**

**Response one:**

Spirit

**Response two:**

Spirit

**Response three:**

Mentholated spirit

**Response:** all affirm spirit

**In which situation are they used?**

**Response one**

Because of the odor

**Response two**

It is used to dry the cord and to avoid infection

**Is the spirit effective in drying the cord?**

**Response one:** yes

**Response two:** yes

**Response**: All mothers affirm yes.

**The problems you mentioned such as bleeding etc. what do you think caused it?**

**Response one:**

The bleeding is that the cord was cut too short, the cord should be long because if it is long it will not bleed

**Response:** All mothers, no proper care.

**What is done before the cord is cut?**

When the baby is born they will first of all clean the cord before enn they cut it

**Is there any special way to bath the new born in this community?**

**Response one**:

Almost all mother's, noooo

**Response two:**

There is, they bath the baby with hot water, Omo and plantain leaf. that is the special way

**The benefits of each method**

**Mother 5**

As for the ‘Never die’ heals the wound.

The alligator pepper is to block air from getting into the cord and make it warm so that the cord will not be infected.

The Spirit helps to dry the cord.

The alligator pepper heals the wound inside the never die help to cut the cord and heal the wound.

**Mother 8**

The ashes cut the cord very fast because in many places, they forbid cord, there are some people that are not suppose to see the child’s cord because when they see it the baby will die, so they use the ashes, salt and the never die to quickly cut the cord.
