# Supplementary material for: Sociocultural practices, beliefs, and myths surrounding newborn cord care in Bayelsa State, Nigeria: A qualitative study
Source: PLOS Glob Public Health. 2023 Mar 28;3(3):e0001299. doi: 10.1371/journal.pgph.0001299 (PMC10047526; doi:10.1371/journal.pgph.0001299)
Supplement: S2 Text — (DOCX) [file pgph.0001299.s002.docx]

**THE KNOWLEDGE PRACTICE AND PREDICTORS OF GOOD CORD CARE AMONG MOTHERS IN BAYELSA STATE**

**FOCAL GROUP DISCUSSION**

**Date of interview: 19/05/2021**

**Start time: 11:45am**

**Stop time: 1:22pm**

**Interviewers name: A.C.S.**

**Note takers name: O.R.E.**

**Interview tool used: I C Sony Recorder**

**Gender of interviewee: Eight Women**

**Location of interview: Tungbo in Sagbama L G A Bayelsa State**

Interviewees accepted to participate in the interview and also agreed to have their voice recorded

**Section one: Demographic information**

**What is your highest level of education, What is your name, How many children do you have, How long have you been in this community etc**

Speaker one: my name is A.A., have been in this community for thirty years and I have four children. I am ---- years old. No education

Speaker two: my name is F.O. I have been in this community for twenty one years now, and I have three kids my last baby is eleven months old.I am ---- years old., OND

Speaker three: my name is P.F. I stay in this community like five years and I have three children the last one is two months,I am ---- years old, secondary

Speaker four: my name is V.. I have been in this community seven years now. My last son is four years old I have two children, I am ---- years old., secondary school

Speaker five: am A.G. I have been in this community six years and I have three children and the last one is just one month. I am ---- years old., primary school

Speaker six: I have stayed in this community two years I have five children my name is N.S., I am ---- years old.

Speaker seven: My name is L , I have been in this community for seven years I have three kids the last one is three months, secondary,I am ---- years old.

Speaker eight: My name is E.G., I have been in this community for one month, I have one child, the last child is four months, I am ---- years old, secondary school

**Where do mother prefer to go to give birth in this community? Is it health center or TBA?**

Respons one: health center,

Response two: health center too,

Response three: No, some people go for massaging home.

Why do they prefer this place? The health center?

Response: one because they feel it is more safer.

**Interviewer the massaging place, why do they prefer to go there?**

Response two: There too safe delivery. Like if you go to the hospital or health centre, they go for operation instead some people go for the massaging home. Money is also involve but some people go because of nearby, it depends when the labour comes, so some people nearby place.

**What are the common cultural practices adopted by mothers when taking care of their newborns?**

Response one: The most cultural practice that we adopts is, the mother that just born won’t come outside up till seven days, apart from that no other one

**Interviewer: where did you deliver your baby?**

Speaker one: massaging home

Speaker two: heath center

Speaker three: massaging home

Speaker four: chemist

Speaker five: massaging home

Speaker six: massaging home

Speaker seven: chemist

Speaker eight: massaging home

**Are there any cultural myths or belief about the newborn cord?**

Response one: No.

Response two: basically the traditional here is that during the first seven days when the child is at home, the mother don’t use to come outside. Even people coming to greet her the mother don’t use to answer most of the greetings you just need to be quite and be looking people because there are many affairs during this period, when you come outside, maybe meet all those people that have those effect when the child navel (cord) have not fall down, when you jam those people outside it normally affects the child inside house, some even lost their lives through those process, may be the child will begin to fall sick, so basic culture is that the first seven days the mother don’t use to come outside.

**Interviewer: who are those people that she is not supposed to see?**

Response one: those people are mostly traditional worshippers because in those days traditional worship is more common than Christianity, so in those days those traditional worshippers that have those laws that don’t use to see any women given birth the first seven day days, if you jam with those people you must surely have effect so that is why they put those laws

**Section two: cord care**

**Right after the baby is born how is the cord cut?**

Response one: they use blade to cut it, they will drag the navel (cord) small before they cut it.

**Who does the cutting?**

Response one: Erezimo ere ( massasing woman)

Response two: the health center is the nurse

**What is usually used to cut the cord?**

Response one: razor blade

**Is anything done before the cord is cut?**

Response one: They will use black thread to tie the cord before they will use blade to cut

Response two: According to my own, they use the peg (cord clamp) to peg the cord and use scissors to cut

Response three: they use rope (black thread) to tie the cord before they cut it with blade

**What is usually used to tie the cord?**

Response one: is the peg

**After birth how is the cord cared for?**

Response one: I use hot water, then I will use native medicine then rob to rob the cord

**Interviewer what is the name of the native medicine**

Response: ombukoromo- diri (never die)

Response two: After birth when I get home I normally use hot water to press the cord, the surface around it and use spirit to clean it then I now cover it properly to prevent air from getting into it until the cord is falling off, I use pepper after cutting it into two and use the water after pressing it to put in the cord. The pepper is the long pepper (tatachi)

Response three: for me I use spirit after I use hot water I use spirit with the cotton wool to clean it. I use rob to rob the corner

Response four: After the cord has falling I use to press it with hot water I put breast milk and I chew alligator pepper and put it inside I use never die also I put never die on fire and press the water inside

Response five: I use alligator pepper with pepper used for cooking and also the leaf never die I boil water to press it, I take rob to rob the cord

Response six: I use spirit use hot water and spirit to clean it after the spirit I use close up rob the corner or I use never die

Response seven: I use the spirit, hot water and close up the red one

**What was reason for using that method you used?**

Response one:the reason behind it was that I learned that it was safe and it will enable the cord to fall off quick

Response two: I used it for the cord to fall quick

Response three: to prevent air from entering inside and for the safety of the baby

Response four: I used it because I want the cord to fall of quick

Response five: to not make the cord to smell and not to rotten

**The method you used does it make the cord to dry or heal faster or separate quickly?**

Response one: it makes the cord to dry and push the cord out to prevent air from entering inside to prevent the baby from getting sick

Response two: to make the cord dry faster and separate faster

Response three: to make it dry and to separate fast

Response four: to make it dry and heal fast

Response five: it makes it to heal and dry fast

**Interviewer; does it make it to separate fast?**

Response one: No it’s a gradual process because it was up to seven days before the cord separate

**Any side effect?**

Response one: it did not affect the baby

Response two: No side effect

Response three: No side effect

Response four: No side effects

Response five: No

Response six: No

Response seven: No

Response eight: No

**The method you used how many days did the cord fall off?**

Response one: three days

Response two: seven days

Response three: my own na too seven days

Response four: four days

Response five: three days

Response six: my own too three days

Response seven: five days

Response eight: five days

**What is usually use to treat or prevent problems of the cord?**

Response one:three; sometimes I use rob to rob it then I normally also use alligator pepper chew it then put the water inside the baby cord

Response two: I use the rob to rob the baby and then chew alligator pepper and put inside the baby cord

Response three I use rob and alligator pepper, chew and put inside

**Why is this method normally used?**

Response one: because is safe , it calms the pain for that moment

Response two: it makes baby feel okay

Response three: because is hot the rob and alligator pepper is hot it won’t let the baby feel the pain again

Response four: the baby will not cry again when I use that method he will now stop

**Of what benefit is it?**

Response one: the cord will not worry the baby

**Interviewer what is the benefits of the native leaf( never die) and all other methods**

Response one: it won’t pain the baby again, it will dry quick, then it will dry quick, the cord will not worry the baby again the rob, after the cord cut I use rob with hot water to treat the baby’s cord the benefits is that it won’t hot the baby cord again

Response two: I use spirit, the spirit helps to dry the cord then alligator pepper, that one help to heal the cord inside then I also use breast milk for the cord to fall fast fall so that air will not enter inside and also for the inside to come outside so that air will not quick enter

Response three: I use salt with close up so that the cord will dry fast I use the salt so that the cord will dry and the cord will not smell and not rotten I also use pepper I use the pepper to put inside water then use the water to put inside cord so that the cord will not pain the baby, then I use breast milk to put inside the cord so that the cord will dry, will not worry the baby and will not smell

Response four: I use spirit because to prevent the cord from smelling, then after I use the spirit I use close up to help the cord to fall fast when the cord cut I use alligator pepper to put inside the cord and rob to prevent the stomach from swelling up

Response five: I use rob with close up I use the rob to prevent the baby’s stomach from swelling up

Response six: I use spirit and close up when I use the spirit to clean the pampu (cord) finish I will use the close up I use the spirit to clean the cord to prevent it from smelling and then I use the close up the red one to rob the cord so that it will dry after the cord has dried it will fall and then I will use rob to put on the cord so that the stomach will not be boiling

**Who taught you this method?**

Response one: my mother

Response two: my mother

Response three: I stayed with my aunt and learned from her

Response four: my mother

Response five: my mother

Response six: my mother

Response seven: my mother

**Interviewer, mention every other place you learned how to use the various methods?**

Response one: my mother, the spirit I learned from hospital but the close up is when I reach home somebody will say that is good because they have used it before, that is why I used it

Response two: I learn from my neighbor to use the close up

Response three: the spirit method I learned from hospital because that was where I born my first born my first child and the close up is some one that told me to use so that the cord will cut fast, it was my mate my husband’s brothers wife

**Does your child’s cord improve or heal better?**

Response one: yes

Response two: yes

Response three: heal better

Response four: yes

Response five: yes good for the baby

Response one: yes

**What kind of problems have you heard about or experienced? Such as bleeding, hotness of the body etc**

Response one: my baby has not experience that kind thing

Response two: me too have not seen that kind thing

Response three: for me sometimes when the baby has cried too much the massaging people will put their hand on the cord and see that the cord is boiling, they will say the cord is paining the baby, like some people they will carry the baby to go sow needle. Traditional way they use to sow it

**Interviewer what do they use to sow it?**

Response: I don’t know what they sow it with, I have not done it, then they use native medicine to rob it.

Response four: they use needle with thread, the buy seven needles they will use black thread and join the needles together and tie until one small place will be left and they will shook the baby with it on the cord and use native medicine to rob the place with kaikai (dry gin) it is for the cord not to pain the baby. apart from this one I have not experience another one

Response five: if I see my baby in pain I will carry him to meet someone and they will say the cord is paining him, if they see it like that they massage him and turn the cord , have not seen another one

Response six: I never see any one apart from this once

Response seven: No

Response eight: I have not experience it, I have not seen it.

**Interviewer; apart from the pain, have seen other signs such as odor, bleeding etc.**

Response one: I have seen some, some will swell up and blood will coming out, they will put spirit inside. I have not seen another one apart from this one

**How common is this problem?**

Response one: yes I have heard of several cases but I don’t know what causes it, but is because they did not treat it fine that is why. When this happen they will inject the baby when they take the baby to hospital but the local one I don’t know that one, they will use rob spirit and that leaf (never die) and put on it.

**What usually happens to the baby?**

Response one: that baby will die

Response two: I have not experienced it but I heard that if the cord is not properly cared for it will make the baby to fall sick

Response three: I don’t have idea

**Have you heard some antiseptics ( such as methylated spirit or chlorhexidine) which are commonly used to clean the infants cords?**

Response one: yes

Response two: I heard that the spirit is good

Response three: yes

Response four: yes

Response five: yes

Response six: yes

Response seven: yes I have heard of the spirit

Response eight: yes

**Which one is mostly used in this community?**

Response one: the spirit

Response two: the spirit

Response three: spirit and never die

**In which situation are they used?**

Response one: as I born the baby and go to the house, I then use the spirit and cotton wool to clean the cord to dry the cord to prevent it from odor

Response two: the spirit is normally, basically is a slow process when you are using the spirit it will delay the cord from falling because is a gradual process , it will make the cord to fall and before the cord will fall even the injury have heal more than the other methods

Response three: it help to dry the water inside yes when you use the spirit to clean the cord it will dry the cord the water inside too will go

Response four: is to dry the cord so that the cord will separate fast, it make the cord to dry fastResponse five: make the cord not to smell

**If you don’t use them why not?**

Response one: Almost everybody here use it

Response : all, yes

**Any side effect for the spirit?**

Response one: NO

**Does Anyone have something important to say about cord care that has not been discussed ?**

Response: all, no other idea
