# Supplementary material for: Sociocultural practices, beliefs, and myths surrounding newborn cord care in Bayelsa State, Nigeria: A qualitative study
Source: PLOS Glob Public Health. 2023 Mar 28;3(3):e0001299. doi: 10.1371/journal.pgph.0001299 (PMC10047526; doi:10.1371/journal.pgph.0001299)
Supplement: S3 Text — (DOCX) [file pgph.0001299.s003.docx]

**THE KNOWLEDGE, PRACTICE AND PREDICTORS OF GOOD CORD CARE AMONG MOTHERS IN BAYELSA STATE.**

**FOCUS GROUP DISCUSSION**

**Date of interview: 08/06/2021**

**Start time: 4:12pm**

**Stop time: 4:47pm**

**Interviewers name: A.C.S.**

**Note takers name: C.D.**

**Interview tool used: I C Sony Recorder**

**Gender of interviewee: Eight Women**

**Location of interview: Ekeki in Yenagoa Local Government Area, Bayelsa State**

**The study is about knowledge, practice and predictors of good cord care among mother's in Bayelsa State**

Interviewees accepted to participate in the interview and also agreed to have their voice recorded

**Section one: Demographic information**

**What is your highest level of education, what is your name, How many children do you have, what is your age, How long have you been in this community etc.**

**Mother one**: my name Is **LO**, I have four children,

ennn for two years now, University

**Mother two:** my name is **S**, three children**,** I am ---- years old., close to fourteen years, primary

**Mother three**: my name is **J**, three children, I am ---- years old, six years, secondary

**Mother four**: my name is **W**, I am ---- years old., four children, ten years, University

**Mother five**: my name is ..., I am ---- years old, one child, eight years, secondary

**Mother six**: my name is **E**, I am ---- years old, two children, ten years. HND

**Mother seven:** my name is **D**, I am ---- years old., two children, for like sixteen years. TC4

**Mother eight:** my names is **A**, I am ---- years old, three children, ten years. secondary

**Where do mother prefer to go to give birth in this community? Is it health center or TBA**

**Response one:**  health center

**Response two:**  health center

**Response three:** mama Ijaw place, that is massaging place

**Response four: massaging,** they like massaging place

**Why do they prefer this place?** The health center?

**Response one:** for unnnn**,** like safety reasons, if you give birth in the health centers they have the facilities then but if you use all this massaging place one can lost their life and baby life

**Why do they prefer the massaging place?**

**Response** one: sometimes they feel is cheaper, sometimes what they believe, that they do more better than the clinic or the health center

**Response two:** at times mama Ijaw because they said mama Ijaw can turn the baby for them to give birth

**What are the common cultural practices adopted by mothers when taking care of their newborns?**

**Response one:** is the normal way, no special way

**Are there any cultural myths or belief about the newborn cord?**

**Response one:** like what I heard that if the oblican cord is still there the baby don’t come out, so that they will not see some certain people, maybe it affect the children I don’t really know, some time they say if they see somebody that is doing traditional medicine, sometimes because the oblican cord is still there that the mother will be having stomach pain

Interviewer: what about the baby? The baby I don’t really know what happen to the baby

**Section two:**

**Right after the baby is born how is the cord cut?**

**Response one:** I think traditionally they use to use thread to tie it, but how they do it before the thread, that I don’t really know.

**Response two: I don't really know oooo**

**Response three:** I hear that they use to use their finger to measure it two times and tie it before they cut it

**Who does the cutting in the health center?**

**Response one:** the mid wife

**The traditional center who does the cutting?**

**Response one:** Is the woman, mama Ijaw, massaging woman

**Where did you give birth, your last baby?**

**Response one:** in the hospital

**Response two:** family support clinic (FSP)

**Response three: Massaging home**

**Response four: FMC Hospital**

**Response five:** FMC hospital

**Response six: FMC hospital**

**Response seven: OKOLOBRI hospital**

**Response eight: Health Center**

**What is usually used to cut the cord?**

**Response one:** scissors

**Response two:** razor blade

**Response three:**  **scissors**

**Response four:** scissors or razor blade

**Interviewer: is anything done to the sessors or blade before it is used.**

**Response one:** if is the clinic they will sterilize it, at times I don’t know what they call it, they will boil it before they use it on the baby’s body

**Is anything done before the cord is cut?**

**Response one:** I don’t see it but they tie it before they clip with clipper (cord clamp)

**Response two:** cord clamp

**What is usually used to tie the cord?**

**Response one:** is cord clip na..

**Interviewer: the massaging home what is usually used?**

**Response one:**  they use thread

**Interviewer: what type of thread do they use?**

**Response one:**  sewing thread

**Response two:**  they use that thread used in plating hair

**After birth how is the cord is usually cared for?**

**Response one:** I use spirit and clean the mouth of the navel (cord), after using the spirit I use mentholator rob and rob it that’s all

**Response two:** after batting the child you clean with a spirit, like me I use saccharin powder then pour it round and then I use bandage to rap the oblican cord with the tommy (stomach) before you wear your dipper eeeee, ennn. Sometimes I add rob. I use spirit saccharin powder, rob and a bandage

**Response three:**  my own I use hot water after, before the cord cut I use spirit between three to two two hours I use spirit to clean it after it has fall I use a separate hot water not the one you her using to bath him, you will not use the one that will burn the child oh but it will be hot so that it will heal the wound, you use towel to touch the hot water and use it to press the navel (cord) after that you now use rob round the cord and the back,

**Interviewer: do you use something’s like close up and never die**

**Response:** no no nooooo

**Response four:** I use spirit to clean it after cleaning it I use rob and massage it inside,

**Response five:** unnnnnnn like me is my mother in-law that use to bath them, after batting the baby she will put dusting powder inside tissue to rap the navel (cord) to now rap it all over the baby with bandage then after when the cord has cut like seven days she will now use hot water to press the novel, at times the baby’s novel(cord) will now be making some kind of noise she will now use alligator pepper, she will shew it with her mouth and I don’t know what else, she will put it in her mouth and be using her month on the cord I don’t know wither she is socking it oh, I don’t know, that’s what she use to do

**Response six**: after bating the baby I will use spirit I will clean it with the spirit and cutton wool the I will use vasline after veslinn I will use close up and I use the close up so that the cord will quick fall

**Response seven:** after batting I use spirit to clean the navel (cord) with spirit then I use rob, put rob then when the cord fall then I use hot water to press it open the cord and use the hot water to press the inside then I use the alligator pepper I shew it and put it inside to make the wound go fast the alligator pepper is to heal the wound and I use it when the cord have fall

**Response eight:**  my own simple as ABC. the spirit and cotton wool…. After the novel (cord) has cut off. I still use spirit and cotton wool that’s all, with the spirit sometimes one week sometimes it depend on the cord, some is thick is between one week some five days so after the thing has cut off I still use the spirit just to clean every time till the cord heal completely, I didn’t use anything else and there was no effect it was normal

**Interviewer: The alligator pepper what was your reason for using it,**

**Response one:** like I said the cord was making some noise and they said it will make it to stop, we use it when the cord as already fall and it stayed for one week to fall that is seven days

**Interviewer: how many days did your baby’s cord fall off?**

**Response one:**  seven days I told you it depend, like if the cord is very thick is like one week but the one that is not that thick in between five days

**Response two:**  seven days

**Response three:** three days

**Response four**: seven days

**Response five**: six days

**Response six:**  five days

**Response seven**: my own stay for two weeks and before the two weeks the place has already heal………. So there was no much wound, and it was very dry because I used the spirit and saccharin powder and also dusting powder so it was not even smelling

**Response eight:**  two weeks, I used spirit and mentholator and there was no side effect and it was dry. I use this method because my first baby I used close up and it fall of quick and before I know air has entered the baby stomach because the cord has not dry, I regretted using it, that’s why I decided to be using spirit

**What is usually use to treat or prevent problems of the cord?**

**Response one:** the spirit just the spirit

**Response two:** the spirit

**Response three**: the spirit and dusting powder to make it dry not to have odor.

**Of what benefit is the spirit?**

**Response one:** emmm is health wise is safe, safer and the way I was thought too and I believe it, using it for my four children it is very very safe.

**Of what benefits is the close up? B it** it makes it to fall quick

**Response two:** most mothers eeen if the thing (ccord) fall quick it qwill be easier for them to bath the baby and do some other things with the baby because with the cord they have to be careful not to dracrk it by mistake, so when the cord fall it become easy to handle th baby, that’s the reason

**Who taught you this method?**

**Response one:** the spirit is hospital, the close up, just m,y neighbor

**Response two:** it was in the hospital I saw the nurse cleaning the cord with the spirit and she told me that when I get home I should be using it

**Response three:** spirit hospital the other methods my mother in-law

**Response four:** the spirit hospital and I use only spirit

**Response five:** my mother in-law

**Response six:** the spirit is from the hospital then the alligator pepper from my mother

**Response seven: all my method is from the spirit** hospital

**Response eight**

**What kind of problems have you heard about or experienced? Such as bleeding, hotness of the body etc.**

**Response one:** because of the use of different treatment like alligator pepper, never die, I don’t know the names they call it but have head stories of children dying, maybe the thing will start smelling, and they will have to take the child to one place they call icu in the hospital that they take care of small chidren, at the end some people even stay there with their babies for three month, four month, so is a very terrible situation, those are the things have heard because of the use of those other methods apart from spirit

**Response two:** like one woman my neighbor, they did not take care of her oblican cord well and she did not even buy spirit, so at a time,,,,, I don’t know what they use the thing(cord) just cut off, before we know what was happening the tommy (stomach) start swelling, like as if air enter into the baby’s stomach and the baby was not able to breath so they took the child to federal medical center (FMC) but the following day the child died. The cord was also very dark and it was smelling.

**How common is this problem?**

**Response one:** anyway, it is very common in some areas but now people find a way to rectify it when they carry the child to hospital

**Interviewer: In this community is cord problems common?**

**Response:** no

**What in your opinion caused those problems?**

**Response one:** I think that the cord was not been taking care of the way it should be

**Response two**. Improper care, as in using too many things like all this alligator and others, I think maybe because the baby’s body can not stand those methods, so all this plenty plenty methods is what cause the problems

**Have you heard some antiseptics ( such as methylated spirit or chlorhexidine) which are commonly used to clean the infants cords?**

**Response one:** yes

**Response two:** yes

**Response three:** yes

**Response four:** yes I have heard about the spirit and saccharin powder

**Response five** yes, spirit and dusting powder

**Response six:**  spirit

**Response seven:**  spirit

**Response eight:**  spirit

**Which one is mostly used in this community?**

**Response one:** the spirit na……

**Response two:** the spirit

**Response three:** spirit

**Response four:** spirit

**Response five:** spirit

**Response six:** spirit

**In which situation the spirit used?**

**Response one**: for the cleaning of the oblican cord

**Response two:** to clean the cord

**Response three**: they use it with cotton wool to clean it

**The spirit, is it effective in drying the cord?**

**Response one:** is very very effective

**Response two:** all, yes…………

**If you don’t use them why not?**

**Response one**: some is ignorant

**Response two**: some they feel is not working for them so they use other things that is working for them

**Response three:** some also feel that it delays the falling of the cord so they want a quick method that will make the cord to cut off in three days’ time, so they use close up and whatever.

**Interviewer: we heard about other methods like salt hashes’ breast milk etc. have you heard of anyone in this community?**

**Response one**: have heard about breast milk

**Response two**: never die also

**Response three:** all, never die yes

**Response:** they roast it and then sguzee out the water and put it inside they said that one works fast, that’s what we heard, laugh.

**Response four**: the never die is a native leaf, they put it on fire, when the leaf is soft they will now sguzze the water or juice to put inside cord to dry it fast….unnnnnn to cut it fast

**Response five:** the breast milk I saw it oh, I saw somebody putting her breast milk inside the cord, but I don’t know weither is to cut it off quick

**Any side effect for the use of the spirit?**

**Response one:** all, no o………..

**Does Anyone have something important to say about cord care that has not been discussed?**

**Response one:** like me oooo if is only me i will advise every mother to use only spirit to clean the cord so that it will get heal without pains

**Response two:** the spirit makes the cord not to have odor but if you don’t use spirit the cord will smell no matter how you think you are batting the baby, mothers should use spirit to clean the oblican cord almost every time, anytime you want to change the dipper, you will never perceive any odor from that child.

**Response three:** this one is advice, this gel chlohexindine is also for the cord, is also like the mentholated spirit because it contains anticeptive, it kills gems too, but this one you don’t need to clean you just apply at this base daily morning and night, on its own it will just heal and just drop off, with the gel you don’t need to use spirit, is either spirit or this gel

**Response**: if you use the gel you don’t need to use spirit?

**Response:** yes, if you use the gel you don’t need to use spirit but many people don’t know about this gel

**Response:** me I use both oooo

**Interviewer: is it wrong to use both?**

**Response**: no is not wrong to use both because both of them are anticeptic and both of them kill gems, but because many women are just used to that cleaning with the spirit so…. when you clean you just apply the gel, is also an anticeptic that prevent gems too and also makes the cord to fall. Thank you.
