# Supplementary material for: Sociocultural practices, beliefs, and myths surrounding newborn cord care in Bayelsa State, Nigeria: A qualitative study
Source: PLOS Glob Public Health. 2023 Mar 28;3(3):e0001299. doi: 10.1371/journal.pgph.0001299 (PMC10047526; doi:10.1371/journal.pgph.0001299)
Supplement: S4 Text — (DOCX) [file pgph.0001299.s004.docx]

**THE KNOWLEDGE, PRACTICE AND PREDICTORS OF GOOD CORD CARE AMONG MOTHERS IN BAYELSA STATE.**

**INDEPTH INTERVIEW TBA**

**Date of interview: 12/05/2021**

**Start time: 11:01am**

**Stop time: 11:46am**

**Interviewers name: A.C.S.**

**Note takers name: O.R.E.**

**Interview tool used: I C Sony Recorder**

**Gender of interviewee: One woman**

**Location of interview: Emayah 2 in Ogbai Local Government Area Bayelsa State**

Interviewee accepted to participate in the interview and also agreed to have her voice recorded

**Section one: Demographic information**

**What is your name Ma?**

**Response: A.U.**

**How old are you?**

**I am......... years old**

**What is your title/designation?**

**Response**: ennnnn massasing woman

**What is your highest level of education?**

**Response:** Intel,,,,,, no..primary

**How long h**

**ave you been in this community?**

**Response:** since I was born

**How long have you been playing the role of Traditional Birth Attendant?**

**Response:** five years now

**Do you attend to a lot of birth in this community?**

**Response:** yes

**What are the common cultural practices adopted by mothers when taking care of their newborns?**

**Response:** unnnn some of them sha, some of them that first time they will put the baby in their lap, they usually stress their legs on the basin and place the new born baby on their leg, then they will use small basin and fetche water and bath the baby on their leg

**Are there any cultural myths or belief about the newborn cord?**

**Response:** na that novel (cord) when you cut it you dig the ground and bury the placenta with it

**Right after the baby is born how is the cord cut?**

**Response**: we usually use never die (native leaf) they will ground it and then place it round the navel (cord) like after four days it will cut

**Interviewer: what do you normally do before cutting the cord?**

**Response:** ok, okkkkkkk, I will first of all cut the cord before cleaning the cord, then I will use razor blade,,,, but I will tie the cord with ennnnnn,,, you will use thread and tie the cord, you will tight it well so that it will not bleed after tying it well you use the razor blade to cut it, when you cut it you put the baby on your laps and start cleaning the baby after cleaning you rap the baby with wrapper after you have wear him everything and then you lie down the baby on the bed

**Who does the cutting?**

**Response:** myself

**What is usually to cut the cord?**

**Response:** razor blade

**Is there any special thing you do before the cutting of the cord?**

**Response:** No...

**What is usually used for the tying of the cord?**

**Response:** Thread

**How do most mothers**

**Response:** they use hot water, and one leaf know as never die and then they will grand it and put it for at list three to four days it will cut, it is now they usually put spirit.

This other side English way they will add the spirit,,, laugh then that’s what I know and alligator pepper when the cord has cut, they put it after batting with hot water, you put it inside the cord, my own side this is the way we usually do, some people they usually cover it because of breeze so that air will not penetrate inside, so they usually cover the cord, they tie it

**Why is this particular method of never die used?**

**Response:** that never die is to make it cut fast, so that it will not stay too long, so after the cord is cut they start applying alligator pepper in that place so that air will not enter inside the tommy (stomach)

**What is the benefit of this method?**

**Response:** So that the baby will be alive na… without using all those things the baby will have sickness or disease na….then the baby will die

**Does it make the cord to heal, dry and separate faster?**

**Response:** it make it dry and separate quickly and heal

**Any side effect?**

**Response:** no,,,, on less you don’t treat it well it will bring out purse if you don’t treat it well it will bring problem for you

**Interviewer: like what type of cord problems have you seen?**

**Response**: like the stomach will be paining the baby, the baby will be having sleepless nights, they will not sleep they will not eat,,,, when you find out that it is from the cord then you treat the baby

**What do mothers do to prevent problems with the baby’s cord?**

**Response:** you clean the cord with ennn spirit, after cleaning with the spirit with cotton wool to clean the cord then after cleaning, then you put the alligator pepper then you cover the cord of the baby

**What is the benefit of this methods?**

**Response:** To save the baby’s life na..

**Does it make it to heal, dry or fall off fast?**

**Response:** yes na..

**Have you heard of any problem when this is down?**

**Response:** if you don’t treat it well it will bring problem for you

**What are the signs of an infected cord?**

**Response**: sometimes this cord ennn it will bring porse, it will rise up, that is swelling just like boils then it will be paining the baby, the mouth of the cord become red, some small bleeding, the baby will cry because it cannot tell you so he keeps crying and will be having sleepless night.

**Sometime new born babies have a problem with the cord stump?**

**Response:** When you cut that cord very short it will give the baby problem it will give sickness like tetanus, you see new born baby having that problem because the cord is too short so it’s important to make it longer, the cutting of the cord does not have size so make it longer but don’t make it shorter

**How common is this problems in this community?**

**Response:**  not so much

**What in your opinion caused the problems you just mention?**

**Response:** cutting the navel(cord) short

**What do you do when this happen?**

**Response:** you carry the baby to the hospital, you can’t handle it by yourself because you don’t know anything concerning the sickness soooo

**What usually happens to the baby with tetanus?**

**Response:** you usually see the baby just stressing his or herself like that, you see them bringing out their tongue then you know problem is coming then you rush to the doctor

**Have you heard some antiseptics (such as methylated spirit or chlorhexidine) which are commonly used to clean the infants’ cords?**

**Response:** yes the spirit

**Which ones are commonly used in this community?**

**Response:**  this days we usually use what you mentioned the mentholated spirit with the cotton wool to clean it this days

**In which situations are they used?**

**Response:** That spirit when,,,,, when you deliver the following day you start using it before the cord will cut you continue using it to be cleaning the cord so that it will not have infection it will dry

**Is it effective in drying the cord?**

**Response:** no, ennnnn when you use it will start drying the cord it will not bring wound and heal quick, it will dry and it will cut off. It make it to dry quick

**For those who don’t use them, any reason why they are not used?**

**Response**: this days there is nobody that don’t use those things, the mentholated spirit

**Mention all the methods mothers her using in this community?**

**Response:** spirit with cotton wool, alligator pepper, never die we apply rob too round the cord, just round the cord you will not put it inside just round, every morning, they use hot water too, they use the hot water before applying all this once have mentioned

**Any unusual side effect of the spirit used?**

**Response:** No oo

**That first day the baby is born how do you bath the baby?**

**Response:** for me that first day I don’t bath the baby I only clean the baby, it’s from the second day and there is no special way just warm water and Dettol.
