# Supplementary material for: Sociocultural practices, beliefs, and myths surrounding newborn cord care in Bayelsa State, Nigeria: A qualitative study
Source: PLOS Glob Public Health. 2023 Mar 28;3(3):e0001299. doi: 10.1371/journal.pgph.0001299 (PMC10047526; doi:10.1371/journal.pgph.0001299)
Supplement: S5 Text — (DOC) [file pgph.0001299.s005.doc]

**THE KNOWLEDGE PRACTICE AND PREDICTORS OF GOOD CORD CARE AMONG MOTHERS IN BAYELSA STATE**

**Indepth interview of TBA**

**Date of interview: 19/05/2021**

**Start time: 1:12pm**

**Stop time: 1:57pm**

**Interviewers name: A.C.S.**

**Note takers name: O.R.E.**

**Interview tool used: I C Sunny Recorder**

**Gender of interviewee: Male**

**Location of interview: Tungbo in Sagbama L G A Bayelsa State**

**Section one: What is your title/designation**?

**Response**: **G.K**., they call me **Dr G.K.**, they are calling me all these names

**How old are you?**

**Response**: I am......... years old

**How long have you been in this community?**

**Response**: from my youth, since them born me. I have been in this community

**How long have you been playing the role of Traditional Birth Attendant?**

**Response**: okay that one, is getting long and I did not take record of when I started it. It has been long, I am now more than seventy two years now, it will be about fifty years now because I started it since my youth, it is God’s gift., nobody thought me, God gave it

**Do you attend to a lot of birth in this community?**

**Response:** they are plenty, this week have not delivered any woman , two weeks ago I delivered one woman and another two weeks ago I delivered one woman from a Orua, this month and last month I have delivered two and some months I deliver three

**What are the common cultural practices adopted by mothers when taking care of their newborns?**

**Response:** there is no any special way

**Are there any cultural myths or belief about the newborn cord?**

**Response:** olden days’ time, when we cut the cord, we use leaf to cut the cord, okay…. if someone that just born when the cord has not falling, when some people come and see the baby the baby will die. Those are the bad people who put bad medicine in their body. You don’t know them but if you born, they will hear and they will near the place so that if they see the woman breast the following day the baby will die, that is the main thing. When the cord drop that thing is finished, then the mother can come out, in the olden days nine days the mother will stay inside the house till the cord done fall but the baby will stay inside. But that thing is no longer there, let me not lie

**Section two: cord care practice**

**Right after the baby is born how is the cord cut?**

**Response:** that one, for me if I have delivered you I will just draw the cord to make the dirt come to the front that is inside, I draw them to the front and tie it with rope , because the foreign people they will use peg to peg, as we don’t have the peg we take rope, clean rope to tie it, and I use new blade that has never been used on anybody else to cut it, blood will not come out again, we use normal blade, yeah no special thing is done on the blade before cutting the cord with it, the rope is the thread that machine sowing thread and the olden days one used for tying hair

**Who does the cutting?**

**Response:** I am the person who do it

**What is usually used to cut the cord?**

**Response:** a blade

**Is there any special thing you do after dragging the cord before cutting?**

**Response:** No. only that one and all the once I have done, there is no report of cord pains the cord is normal the baby will grow .

**What is usually used to tie the cord?**

**Response:** rope (thread) but if the rope is not available any type of thing , if she deliver in the both even cutlass is used to cut the cord, if she born inside canoe she born anything that can be used to cut it and to tie it so that blood will not come out, you tie it very well. Because if you wait for blade the baby will die, the placenta will shoot out and the baby will die

**How do mothers care for their newborn cord immediately after birth?**

**Response:** When you cut the cord finish, now that modern life has come you will use spirit to do it. When you take hot water, without putting the water inside, you just use the towel touch the water, dry it and press it and use spirit to clean the cord, apart from that one you will take breast milk to put inside the cord, the native leaf, alligator pepper too so that the cord will not pain the baby. Never die na strong medicine o.

**Why is this particular method used?**

**Response:** It is God that thought us, the never die if you just put it in fire when you cut it, or put it in lamp that is hot, when you put it until the body gets soft, you squeeze it, not too hot and put it on the cord small time the cord will cut, the alligator pepper is not to allow the cord to pain the baby they use rob too, the rob they use the rob to prevent it from getting pains too, they use pepper for cooking to relieve the vein so that the cord will not pain the baby, the spirit too is to cut the cord quick, it dries the cord, so small time it will cut

**Does it make the cord to heal, dry and separate faster?**

**Response:** Dry and cut quick those two things are guaranteed. It works too for healing, that is the spirit

**What do mothers do to prevent problems with the baby’s cord?**

**Response:** At times some of the cord has healed, it still pain and make some strange noise. There leaves are used to rob it and the problem will be solved. Some are put on fire the native pot, you will put the leaves inside the native pot and put it on fire, when the pot is hot then you drop it on top something and put the leaves and add native pomade (kanel pomade) a little and rob the leaves on the native pomade bit by bit and use it to press the cord, that sickness will be gone. The native pot must not touch the ground. Because of the medicine it must not touch the ground

**What are benefits of this?**

**Response:** To relieve the pain, it will not pain the baby again I use this method after the cord has dropped to prevent any form of pain

**Have you heard of any problem for the infant when this is done?**

**Response:**  No he the medicine they work, the one that I did not deliver also come for the treatment when the cord is paining the baby I have the medicine

**What symptoms of an infected cord have you seen or heard about, such as bleeding etc**

**Response**: they have not brought that type to me before, I have treated only the one that is paining the child

**How common is this problems in this community?**

**Response:** It is not common

**What usually happen to the baby with an infected cord?**

**Response:** They will be crying. The mother will not have rest of mind, constant crying until the problem is solved, the baby body will be hot when they come with those problems, some will complain that the baby cannot sleep throughout the night , at times it is not the cord but hernia, after i massage the baby, after three days of spraying the medicine the problem is gone and the baby will be healed

**Have you heard some antiseptics (such as methylated spirit or chlorhexidine) which are commonly used to clean the infants’ cords?**

**Response:** Yes they are using spirit to look 0clean the baby’s cord, not too common but people are using it. The methylated spirit alligator pepper, pepper and rob and never die, the never die is mostly used more than the rest, they use ashes and salt to make the cord fall quick in three days. The quick falling of the cord sometimes affect the baby and sometimes does not

**In which situations are they used?**

**Response:** all what I know is that the spirit dry the cord, to cut the cord quick and to prevent infection

**Is the spirit effective in drying the cord?**

**Response:** Yes

**For does who don't use them, any reason why they are not used?**

**Response:** some don’t use the spirit because of lack of money to buy it, so they prefer the native ones.

**Any side effects of their use?**

**Response:** No mother has reported any side effects from the spirit
