# Supplementary material for: Sociocultural practices, beliefs, and myths surrounding newborn cord care in Bayelsa State, Nigeria: A qualitative study
Source: PLOS Glob Public Health. 2023 Mar 28;3(3):e0001299. doi: 10.1371/journal.pgph.0001299 (PMC10047526; doi:10.1371/journal.pgph.0001299)
Supplement: S6 Text — (DOCX) [file pgph.0001299.s006.docx]

**THE KNOWLEDGE, PRACTICE AND PREDICTORS OF GOOD CORD CARE AMONG MOTHERS IN BAYELSA STATE.**

**INDEPTH INTERVIEW TBA**

**Date of interview: 08/06/2021**

**Start time: 5.00pm**

**Stop time: 6.00pm**

**Interviewers name: A.C.S.**

**Note takers name: C.D.**

**Interview tool used: I C Sony Recorder**

**Gender of interviewee: One woman**

**Location of interview: Ekeki in Yenagoa Local Government Area, Bayelsa State**

**The study is about knowledge, practice and predictors of good cord care among mother's in Bayelsa State**

Interviewee accepted to participate in the interview and also agreed to have her voice recorded

**Section one: Demographic information**

**What is your title/designation?**

**Response:** The title wey them use to call me is mum, but some the community leader, woman chief of this community, for delivery side they call me mum. Everybody both adult they see me as mother, so they call me mum

**What is your highest level of education?**

**Response:** Well,,,,,secondary but I didn’t finish

**How long have you been in this community?**

**Response:** Since from birth, am from this community, I was born here and I married here

**How long have you been playing the role of Traditional Birth Attendant?**

**Response:** Since 19…………..1998; I am......... years old

**Do you attend to a lot of birth in this community?**

**Response:** Very well, even now self you can see another person here again, this one is not the other one you saw. I have delivered up to four this week, and in a month it depend because some month people use to deliver more than some month, some month you will not see up to three people but some month is up to ten

**What are the common cultural practices adopted by mothers when taking care of their newborns?**

**Response:** We don’t have any special tradition for little born or little baby

**Are there any cultural myths or belief about the newborn cord?**

**Response:** the story I have heard concerning this cord…………… before some people use to born even in there farm so they usually use cutlass or anything that they can use to cut the cord. Yes there are people that are not supposed to see the cord, let me tell you the story, there is a man in this community but I will not name the person,,,,, and the man has three wife and one of the wife have that same problem, you understand? so any time they deliver the baby has they see the placenta or see the new born baby the baby will die, so this thing have been repeating itself until all the eight children he delivered, all died and the woman for the first time thought is the husband that has that problem but she never knew even she has that same problem, that she is not supposed to see the new born, so till the eight child died and as she begin to go to places they told her she too have that problem before she knew that she too have that problem but she hide it from her husband, so the last child they cover her eye, tie her eye so that she will not see the baby and somebody took that side away from her for two weeks, till before they brought the child back to her, that’s the only son that she have, the boy standing here too, I think he knows the story. Apart from this one I don’t know any other one.

**Right after the baby is born how is the cord cut?**

**Response:** Hnnnnn but like as we do for native way, English call am cord clamp but I don’t use to use that one because I don’t know it very well, I use the normal thread that they use to sew cloth either white or black, clean one then between the stomach and the cord I measure two or two and a half inch then I will tie it and cut, no other special thing is done before the cutting except that I tie it three places so that blood no go pour out from the placenta, why I tie two place is in case one is lose the other one will still hold the cord

**Who does the cutting?**

**Response:** I cut it by myself

**What is used to cut the cord?**

New razor blade, the tiger or any other normal razor blade

**Interviewer:** is there any special thing down with the razor blade before cutting it.

**Response**: no special thing is done but sometimes I use cotten wool and if there is spirit I use the spirit and cotton wool

**Is there any special thing you do before the cutting?**

**Response:** No

**What is usually used for the tying of the cord?**

**Response:** Thread

How do most mothers

**Response:** Hmmm……. We as native after...........when somebody deliver in the morning I only clean the baby, in the evening I use spirit to clean the cord, for me I use close up after I use the spirit, the close up is the red close up and rob it round the navel (cord) for three days. The mother’s dey use spirit and this very close up.

**Interviewer, do they use substance like alligator pepper and never die?**

**Response**: No ooh, people use to use it even me I use to use it but is not good, so among all the things have used this two is the best and that’s what I advise all the women to use and when the cord as fall, they use rob because the alligator pepper is too hot and it usually make the navel (cord) to bleed so we use only rob and cover it very well

**Why is this particular method used?**

**Response:** The spirit is,,,, if there is any gem the spirit will clean it off so that the baby will not have tetanus, so we use it to prevent the baby from having tetanus after that we use the close up, and the close up eeeen is to cut it.

**What is the benefit of this method?**

**Response:** the benefit is that, before people people use to use never die and different things and e they result to convotion but since we start using this very pattern no child has ever getting the problem of convotion this is the benefit

**Does it make the cord to heal, dry and separate faster?**

**Response:** On the spirit, makes it dry but not separate it quick, the close up separate it quick within three or four days

**Interviewer: why do you want the cord to separate quick?**

**Response:** the reason why we do so, like my second daughter they advise me not to use any other thing that I should use only spirit. I use only spirit for eight days it did not separate the cord and my daughter was smelling and even when visitor comes I will be ashamed of my baby that is why we use close up to separate and heal the cord fast

**Any side effect?**

**Response:** no side effect

**What do mothers do to prevent problems with the baby’s cord?**

**Response:** is it when the cord as not cut, yes, that time you don’t expose it to breeze so after doing so you cover it immediately so that breeze will not enter the child belly ( stomach ) so that the stomach will not swell or different problem so……… na by covering………… by covering the navel (cord) apart from this one I don’t know any other way

**What is the benefit of this method?**

**Response:** the benefit is to prevent the breeze because if breeze enter the stomach the child will have this sickness, English people call it wither na nimoniar, that will enter the child body all this place rips will be stiff and the stomach will rise that’s the reason we cover the child cord because there is a wound in the cord and through that wound breeze can enter inside the belly (stomach) and the child will have problem

**Have you heard of any problem when this done?**

**Response:** I never here of any problem

**Sometime new born babies have a problem with the cord stump?**

**Response**: unnnnnn the one that have experience is the one they call cord clamp, I said let me try it, Then I begin to use it, as I use it, I see that some of the cord is bigger than some cord some is slim some is fat, those fat once as I use the clamp clip, I see that there is licage and blood will drop from that place, that is the reason why I don’t use it, the cord clamp. I use thread to tie because since have been using the thread no body done complain

**Interviewer: I will like to know the problems such as odor, bleeding etc**

**Response:** unnnnnnnn I done here of purse own and bleeding, the swelling of stomach have heard from people but I have not seen, hotness of the body, the child getting cold, all this once we they here that’s why we use the method to do it fast so that make those other treatments no result to those problems wey we they hear

**How common is this problems in this community?**

**Response:**  is not common again

**What in your opinion caused the problems you just mention?**

**Response:**  na…… when them no treat and them no take good care of the cord it can result to those once. But secondly I get something to tell you, normally when this outside of the cord don heal the inside as not heal, you understand? the inside never heal na from three months before both inside and outside is ok for the child, that’s why we use hot water even though the outside has heal we use hot water to press the stomach of that child so that stomach will not be strong and the baby will go to toilet frequently so that the stomach will not pain the child because if it pains the baby the baby will be shaking and restless, so you know that is the cord that is paining the baby the inside have not heal, nnnnnnn so after the hot water we use rob and we as native people we have many ways of doing it, for me when the baby don (Is) doing that way, you know that the inside is not heal. So I have one native medicine I use dey………… when I have mix it, I wash the leaf and mix it finish, I mix it with hot water too then I use to carry pump this pump that they sell in the market and put it through the annues just little then I will cover the annues, shack the belly (stomach) the baby will seat (toilet) out all those dro dro, slippery green things those things that makes the child stomach to bite as he seat (toilet) out those things that was all… that shacking will stop

**What usually happens to the baby?**

**Response:** I told you that if they don’t treat the cord well… it can lead to tetanus from tetanus to convortion and convotin cause death so because of that, we take good care of that cord

**Have you heard some antiseptics (such as methylated spirit or chlorhexidine) which are commonly used to clean the infants’ cords?**

**Response:** me I use methylated spirit

**Interviewer: What about the chlohexidined?**

**Response:**  I never here that one o

**Which ones are commonly used in this community?**

**Response:**  is this methylated spirit that we use

**In which situations are they used?**

**Response:** we use it to clean the cord even that initial early) that the cord cut we still use the spirit to clean so that gem will not enter. I see the spirit as something that is used to prevent the baby from infection of the cord that’s what I see

I**s it effective in drying the cord?**

**Response:** unnnn yes it dries the cord

**For those who don’t use them, any reason why they are not used?**

**Response:** everybody have their own mind, maybe they like their own method more, make he they petepete soft (easy)

**Interviewer: some mothers said is because of money**

**Response:** unnnn some mothers sometimes will not have the money to buy because even here that some come to deliver, some no get money to pay me, we do the delivery for them in the name of God, so some no get the money to buy. This is the reason I know

**Any unusual side effect of the spirit used?**

**Response:** No get any side effect
