# Supplementary material for: Sociocultural practices, beliefs, and myths surrounding newborn cord care in Bayelsa State, Nigeria: A qualitative study
Source: PLOS Glob Public Health. 2023 Mar 28;3(3):e0001299. doi: 10.1371/journal.pgph.0001299 (PMC10047526; doi:10.1371/journal.pgph.0001299)
Supplement: Attachment — Submitted filename: CORRECTED RESPONSE TO REVIEWERS.docx [file pgph.0001299.s007.docx]

**RESPONSE TO EDITOR**

1. We ask that a manuscript source file is provided at Revision. Please upload your manuscript file as a .doc, .docx, .rtf or .tex.

**RESPONSE: DONE**

2. In the online submission form, you indicated that "The data set for this study is available to be shared on request." All PLOS journals now require all data underlying the findings described in their manuscript to be freely available to other researchers, either 1. In a public repository, 2. Within the manuscript itself, or 3. Uploaded as supplementary information.This policy applies to all data except where public deposition would breach compliance with the protocol approved by your research ethics board. If your data cannot be made publicly available for ethical or legal reasons (e.g., public availability would compromise patient privacy), please explain your reasons by return email and your exemption request will be escalated to the editor for approval. Your exemption request will be handled independently and will not hold up the peer review process, but will need to be resolved should your manuscript be accepted for publication. One of the Editorial team will then be in touch if there are any issues.

**RESPONSE:**

**THE DATA SET FOR THIS STUDY HAS BEEN UPLOADED AS SUPPLEMENTARY INFORMATION. IT INCLUDES THE TRANSCRIBED NOTES OF THE IN-DEPTH DISCUSSIONS FROM THE 3 TRADITIONAL BIRTH ATTENDANTS AS WELL AS THOSE FROM THE PARTICIPANTS OF THE FOCAL GROUP DISCUSSIONS. IT CAN BE MADE PUBLIC AS WRITTEN CONSENTWAS OBTAINED FROM ALL THE PARTICIPANTS.**

3. Please review your reference list to ensure that it is complete and correct.

**RESPONSE: DONE**

**RESPONSE TO REVIEWERS**

**REVIEWER 1**

Reviewer #1: Congratulations on your paper! You have done a great work!

Understanding cultural beliefs about cord clamping and care requires background and social knowledge. Choosing to conduct a qualitative study was a good way to present these groups practices and how they are influenced by elder people. The use of threads, leafs, balms, toothpaste and ashes is common in other poor countries, what explains the high rate of avoidable neonatal deaths. To interview and audiotape the sessions was clever. Minimizes comprehension issues the participants could have.

There is one major issue on supporting information session: the interviewees said their names at the beginning of the meetings. You should suppress this information, by using the designated identification written in the paper.

Minimal issues: some typos on discussion section.

Therefore, your paper is well written, refers to a common problem in deprived places and employs a smart approach to access interviewees opinions.

**RESPONSE TO REVIEWER 1**

Thanks for your kind comments about our paper. Concerning the comments on the supporting information, we obtained written consent from the mothers prior to conducting the study. The reason for giving their names was to create rapport and a relaxed atmosphere during the discussion. Their names were also anonymized before inclusion of their comments in the manuscript.

Typos in the discussion have been checked,

**REVIEWER 2**

Reviewer #2: The paper is interesting. It highlights a topic that is very important.
Recommendations

Introduction
Add statistics concerning the complications related to the use of harmful practices. This is important to show evidence about the magnitude of the problem.
Situate a bit more the study in its context. Reflect on the healthcare system; maternal and child care; percentage of home deliveries and why; etc.
Mention the gap; what is missing.

Methods
Identify the criteria considered for the purposeful sample.
How many women in each FG?
Explain more the plan of analysis.
How did you ensure rigour?
Did you have to translate the language? If yes, how did you ensure accuracy of the meaning?

In the conclusion, add recommendations that make the healthcare system more accessible, affordable, and acceptable for pregnant families.
Edit English.

**RESPONSE TO REVIEWER:**

**Introduction:** The magnitude of the problem has been stated in the last line of the first paragraph of the introduction and beginning of the second paragraph. More statistics have been added concerning the health care system in Bayelsa State which represents what is seen in Nigeria as a whole. The gap which this study aims to address has also been added.

**Methods**

The participants for the Focal group discussions and the In-depth Interviews were recruited by the women leader of each community based on their ability their willingness to participate in the study as well as their vocal ability.

There were 8 women in each FGD.

Analysis of this qualitative data was done using Thematic analysis, through the inductive model using NVivo QSR version 12.2 Pro.

We ensured rigour by having a research question which we believe was answered through our findings. “This study was thus conducted to describe the various types of cord care practises employed by women in Bayelsa State and to explore the cultural beliefs and myths facilitating their adoption”.

We used “pidgin’ English to conduct the interviews which was translated to English by a local interpreter.

**Conclusion**

The suggestions have been added.
